# Tai Chi Chuan in Medicine and Health Promotion

**DOI:** 10.1155/2013/502131

**Published:** 2013-09-12

**Authors:** Ching Lan, Ssu-Yuan Chen, Jin-Shin Lai, Alice May-Kuen Wong

**Affiliations:** ^1^Department of Physical Medicine and Rehabilitation, National Taiwan University Hospital, 7 Chung-Shan South Road and National Taiwan University, College of Medicine, Taipei 100, Taiwan; ^2^Department of Physical Medicine and Rehabilitation, Chang-Gung Memorial Hospital and Department of Physical Therapy, Post-Graduate Institute of Rehabilitation Science, Chang-Gung University, Taoyuan 333, Taiwan

## Abstract

Tai Chi Chuan (Tai Chi) is a Chinese traditional mind-body exercise and recently, it becomes popular worldwide. During the practice of Tai Chi, deep diaphragmatic breathing is integrated into body motions to achieve a harmonious balance between body and mind and to facilitate the flow of internal energy (Qi). Participants can choose to perform a complete set of Tai Chi or selected movements according to their needs. Previous research substantiates that Tai Chi has significant benefits to health promotion, and regularly practicing Tai Chi improves aerobic capacity, muscular strength, balance, health-related quality of life, and psychological well-being. Recent studies also prove that Tai Chi is safe and effective for patients with neurological diseases (e.g., stroke, Parkinson's disease, traumatic brain injury, multiple sclerosis, cognitive dysfunction), rheumatological disease (e.g., rheumatoid arthritis, ankylosing spondylitis, and fibromyalgia), orthopedic diseases (e.g., osteoarthritis, osteoporosis, low-back pain, and musculoskeletal disorder), cardiovascular diseases (e.g., acute myocardial infarction, coronary artery bypass grafting surgery, and heart failure), chronic obstructive pulmonary diseases, and breast cancers. Tai Chi is an aerobic exercise with mild-to-moderate intensity and is appropriate for implementation in the community. This paper reviews the existing literature on Tai Chi and introduces its health-promotion effect and the potential clinical applications.

## 1. Introduction

**Figure 1:**
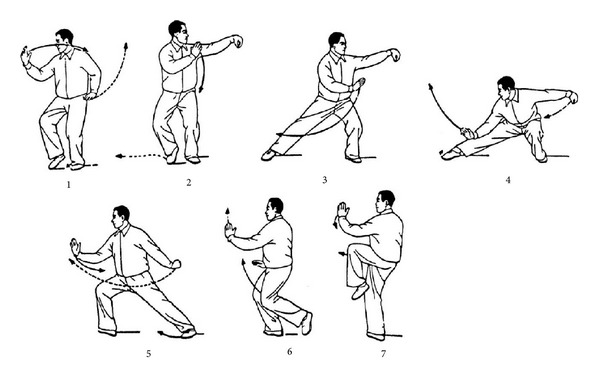
An example of a typical form of Tai Chi (push down and stand on one leg). The sequential motions are performed in a semi-squat posture. (From [1], with permission).

Tai Chi Chuan is a branch of Chinese martial arts and has developed since the 17th century in China. The slow, supple, and graceful movement of Tai Chi is rooted in Taoism. Taoism is an ancient Chinese philosophy and has been taught by Lao Tze in the 5th-4th century B.C. The Taoist doctrine is focused on mind tranquility, and its goal is to achieve longevity by meditation and lifestyle modification. In the process of development, Tai Chi differentiated into five main styles: Chen, Yang, Wu (Hao), Wu, and Sun [1]. Among them, Chen style is the oldest, while Yang style is the most popular. The classical Tai Chi styles consisted of complex forms, and they take long time to learn and practice. Therefore, many simplified Tai Chi forms were developed to shorten the learning period. Variations in training approaches result in significant differences in exercise intensity and training effect. Tai Chi is performed in a semisquat position (Figure 1), and the exercise intensity can be easily adjusted by controlling the speed and postural height. The characteristics of Tai Chi include (1) mind concentration with breathing control, (2) whole-body exercise in a semisquat posture, and (3) continuous, curved, and spiral body movements [1]. Tai Chi can be practiced alone or as a group exercise, and it has significant benefits for physical, emotional, and social functions. Participants may practice several Tai Chi movements instead of a whole set to achieve specific health benefits, such as flexibility and balance. However, if they want to enhance aerobic capacity or muscular strength, a complete set of classical Tai Chi is recommended. In the recent years, Tai Chi has become a popular exercise worldwide, and researches are flourishing. The objective of this paper is to review the existing literature on Tai Chi and to introduce the characteristics of training (exercise intensity and biomechanical aspects), the effect on health promotion (aerobic capacity, muscular strength, balance, health-related quality of life and psychological well-being), and potential applications in medicine (e.g., neurological diseases, rheumatological diseases, orthopedic diseases, cardiopumonary diseases, and cancers). 

## 2. Training Characteristics of Tai Chi

### 2.1. Exercise Intensity

**Figure 2:**
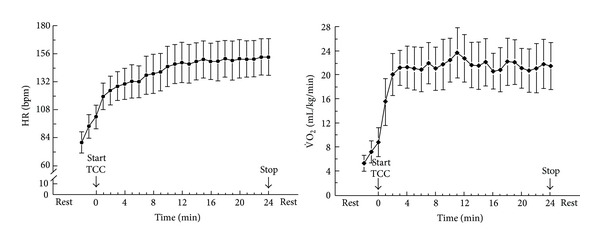
Heart rate response and oxygen uptake during the practice of classical Yang Tai Chi in middle-aged men (values are mean ± SD) [2].

**Figure 3:**
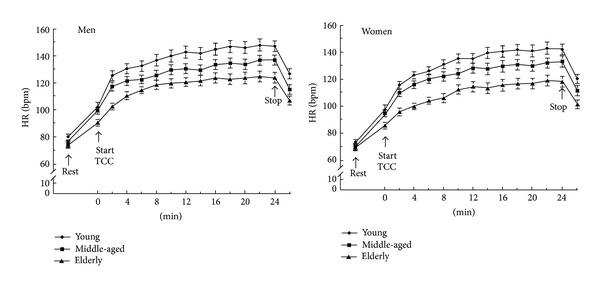
Heart rate responses of men and women during the practice of classical Yang Tai Chi in different age groups (◆ young group, ■ middle-aged group, and ▲ elderly group; values are mean ± SE) [4].

The exercise intensity of Tai Chi depends on its training style, posture, and duration. Variations in training approaches result in substantial differences in exercise intensity. Lan and colleagues [2] have measured heart rate (HR) responses and oxygen uptake while performing classical Yang Tai Chi in middle-aged subjects.  Figure 2 illustrates the heart rate response, and oxygen uptake ($\dot{\text{V}}{\text{O}}_{2}$) during the practice of Tai Chi. In the 24 minutes of practice, subjects' HR increased rapidly in the first 12 minutes and then increased slowly towards the end of the exercise. By contrast, subjects' $\dot{\text{V}}{\text{O}}_{2}$ showed a sharp increase in the first three minutes, and then it achieved a steady state towards the end of the exercise. In the steady state of Tai Chi practice, the average HR was 58% of the heart rate reserve (HRR), and the oxygen uptake was 55% of the peak oxygen uptake ($\dot{\text{V}}{\text{O}}_{2\text{peak}}$). HRR is the difference between maximum heart rate and resting heart rate. HRR is typically utilized to establish HR-based training zones according to the heart rate reserve method. The HRR method is demonstrated as follows: Target HR = [(HR_max⁡_ − HR_rest_) × % intensity desired] + HR_rest_. According to the recommendations of American College of Sports Medicine, moderate-intensity (40%–59% of HRR) aerobic exercise is recommended for most of the adults [3]. The HR during Tai Chi practice is 50%–58% of HRR in subjects aging from 25 to 80 years (Figure 3), which indicates that the exercise intensity is similar across different ages [4]. Previous studies reported that the energy cost during Tai Chi practice was between 3 and 6 metabolic equivalents (METs) depending on different styles and training requirements. Therefore, a suitable style of Tai Chi and selected movements can be chosen to fit participants' needs.

### 2.2. Biomechanical Aspects

Wu and Hitt [5] have examined the kinematics of Tai Chi gait (TCG) and normal gait by using a motion analysis system and biomechanical force plates. TCG had a low-impact force, an evenly distributed body weight between the fore-foot and the rear-foot, and a large medial-lateral displacement of the foot center of pressure (COP). The low-impact force may be attributed to the slow speed of Tai Chi and the coordinated muscular activities of the lower extremities. The activation duration of leg muscles, especially the knee extensors, is significantly affected by the speed of Tai Chi movement. Practicing Tai Chi at a different speed may alter the role of muscular function in movement control [6]. Additionally, the spatial, temporal, and neuromuscular activation patterns of TCG were different with normal gait. Compared with normal gait, Tai Chi gait had (1) a longer cycle duration, and duration of single-leg stance; (2) a larger joint motion in ankle dorsi/plantar flexion, knee flexion, hip flexion, and hip abduction; (3) a larger lateral body shift; and (4) a significant involvement of ankle dorsiflexors, knee extensors, hip flexors and abductors, longer isometric and eccentric actions, and longer coactivations of muscles [7]. Normal muscle activation patterns are characterized by activation and relaxation related to the agonist and antagonist muscle groups during a specific activity. Coactivation of muscle groups is a common strategy adopted to reduce strain and shear forces at the joint [8].

Age may affect the characteristics of Tai Chi performance. The elderly people practice Tai Chi in a higher posture because of muscle weakness or degeneration of knee joints [9]. Tai Chi gait has an increased shear force and frontal plane torque at lower extremity joints than normal gait, but the shear force at lower extremity joints during TCG is lower in the elderly subjects than in the young adults [10].

### 2.3. Cost

Tai Chi is a low-cost exercise because equipment and facility are not needed. In the Chinese community, most of the instructors are volunteer, and participants only need to pay minimum tuition fees. In the United States, a study [11] reported that the direct cost of a Tai Chi program was about $3.5 per person per session, and the cost was affordable for most participants. In Taiwan, a formal Tai Chi training course for novice participants usually costs $20–40 per month. In a recent review to evaluate the strategies to prevent falls among older people [12], Tai Chi was the most cost-effectiveness strategy to prevent falls. 

## 3. Tai Chi for Health Promotion 

### 3.1. Aerobic Capacity

The peak oxygen uptake is the best indicator for aerobic capacity and is the strongest predictor of the risk of death among normal subjects and patients with cardiovascular diseases [13]. In cross-sectional studies, Lan and colleagues [14] have reported that elderly Tai Chi practitioners showed 18%-19% higher in $\dot{\text{V}}{\text{O}}_{2\text{peak}}$ than their sedentary counterparts. Furthermore, long-term Tai Chi practitioners displayed slower age-related decline of aerobic capacity than sedentary individuals. In a five-year follow up study [15], the annual decrease of $\dot{\text{V}}{\text{O}}_{2\text{peak}}$ in the Tai Chi group was about 40% slower than in the sedentary control group. Lan and colleagues [16] also reported that the $\dot{\text{V}}{\text{O}}_{2\text{peak}}$ increased 16.1% and 21.3% after one year of Tai Chi training in older men and women, respectively. According to a recent meta-analysis [17], practice of Tai Chi may significantly improve aerobic capacity. Middle-aged and older women and men benefit the most, with greater gains seen among those initially sedentary. 

### 3.2. Muscular Strength

Tai Chi is performed in the semisquat position, and various degrees of concentric and eccentric contractions are demanded in this unique posture. In the Frailty and Injuries: Cooperative Studies of Intervention Techniques (FICSIT) study [18], Tai Chi program might preserve the strength gains from a 3-month strength training program using instruments, and significant gains persisted after 6 months of Tai Chi. 

Twelve to 24 weeks of Tai Chi exercise appears to be beneficial to muscular strength of lower extremities. Jacobson and colleagues [19] reported that the 12 subjects aging 20–45 years who performed 108-form Tai Chi three times per week for 12 weeks significantly increased the muscular strength of their knee extensors. Lan and colleagues [20] found that Tai Chi exercise enhanced strength of knee extensors at various angles. After 6 months of Yang Tai Chi training, men increased 13.5–24.2% of isokinetic strength in concentric contractions and increased 15.1%–23.8% in eccentric contractions. Wu and colleagues [21] also reported that Tai Chi participants had higher concentric and eccentric strengths of knee extensors and smaller foot center of pressure excursions in both eyes-open and eyes-closed conditions than the controls. The degree of knee flexion during single-leg stance of Tai Chi may be a key element for improving leg muscle strength [22]. In a recent study, Lu and colleagues [23] measured muscular strength of knee by isokinetic testing at 30°/s. The Tai Chi group demonstrated greater eccentric muscular strength in both knee extensors and flexors than the control group.

In elderly individuals, Li and colleagues [24] reported that a 16-week Tai Chi program increased 19.9% of muscular strength of the knee flexors, and there was a significant decrease in latency of semitendinosus muscle in the Tai Chi group. The prevention of falls depends on the timely initiation of an appropriate postural response. Tai Chi intervention significantly hastened the reaction time of the semitendinosus muscle, which may help older people maintain postural control. In a recent randomized trial, a 16-week Tai Chi program three sessions per week also induced a significant increase in eccentric knee extensor strength in senior female subjects [25]. 

### 3.3. Balance and Motor Control

Standing balance is a complex process that depends on the integration of mechanical, sensory, and motor processing strategies. The visual, proprioceptive, and vestibular systems are three sources of afferent information to influence the control of balance, which is termed “sensory organization” [26]. The sensory organization testing (SOT) can be used to identify problems with postural control by assessing the subject's ability to make effective use of visual, vestibular, and proprioceptive information.

During the performance of Tai Chi, weight shifting, body rotation, and single-leg standing in different positions are frequently practiced. Delicate joint control with muscle coordination is required during motions, and hence balance function may benefit from long-term practice of Tai Chi. In studies using simple balance tests (e.g., time duration in single-leg standing with eyes open or closed), older Tai Chi practitioners showed better postural control than sedentary subjects [27, 28]. In a study using computerized balance system, Tai Chi practitioners showed no difference compared to control group in simple conditions (such as postural sway in standing with eyes open or close) [29]. By contrast, Tai Chi participants showed better performance in complex conditions, such as eyes closed with sway surface, sway vision with sway surface, and forward-backward weight shifting test [29]. Many studies have demonstrated the advantages of Tai Chi on visual, proprioceptive, and vestibular functions, and they are described briefly below. 

#### 3.3.1. Visual System

In elderly people, Tai Chi participants had better postural stability at the more challenging condition of sway-referenced vision and support than the control group [29]. Tsang and colleagues [30] investigated elderly Tai Chi practitioners using the SOT and found that their visual ratio was higher than that of nonpractitioners, and even comparable with that of the young subjects. The results implied that long-term practice of Tai Chi improved balance control in the elderly population, and there was an increased reliance on the visual system during stance. Additionally, elderly Tai Chi practitioners attained the same level of balance control as young subjects when standing in reduced or conflicting sensory conditions. In a recent study, Chen and colleagues [31] investigated the effects of Tai Chi for elderly persons with visual impairment and found that the Tai Chi group showed significant improvements in visual and vestibular ratios compared with the control group.

#### 3.3.2. Proprioceptive System

Tai Chi training puts a great emphasis on exact joint positions, and it may improve the sense of position of lower extremities. Wong et al. [32] and Tsang and Hui-Chan [33] examined the knee proprioception in elderly subjects by using the passive knee joint reposition test and found that Tai Chi practitioners had better knee joint proprioceptive acuity than control subjects. In another study, Tsang and Hui-Chan [34] reported that both Tai Chi practitioners and golfers had better knee joint proprioceptive acuity than the elderly control subjects, and it was similar to that of the young subjects. Similarly, Xu and colleagues [35] reported that Tai Chi participants not only showed better proprioception at the ankle and knee joints than the controls, but they also showed better ankle kinesthesis than swimmers/runners. 

Training duration of Tai Chi may influence the accuracy of joint position sense. Fong and Ng [27] have compared long-term (practice for 1–3 years) and short-term (practice for 3 months) Tai Chi training for middle-aged and older individuals. The results showed that both long-term and short-term Tai Chi training improved joint position sense, but only long-term practice could enhance dynamic standing balance. 

Tai Chi also improves proprioceptive function of upper extremities. Tai Chi practitioners focus specific mental attention on the body and upper extremities, which may facilitate tactile acuity and perceptual function. Previous study showed that Tai Chi training could increase shoulder kinesthetic sense and reduce movement force variability in manual aiming tasks. Recent study also found that Tai Chi practitioners attained significantly better eye-hand coordination in finger pointing than control subjects [36].

#### 3.3.3. Vestibular System

Elderly Tai Chi practitioners had better maximal stability and average velocity than the controls under the condition of eyes closed and sway-referenced support (ECSS), which indicated improvement of balance function through vestibular mechanism [32, 37]. Practicing Tai Chi involves head movements and thus stimulates the vestibular system. Therefore, the elderly Tai Chi practitioners could attain a higher vestibular ratio than the controls under the condition of ECSS.

Patients with dizziness and balance disorders may get benefits from Tai Chi training. Hain and colleagues [38] reported that patients with dizziness who practiced 8 Tai Chi movements every day for at least 30 min showed significant improvements in the SOT and the Dizziness Handicap Inventory scores. McGibbon and colleagues [39] found that both Tai Chi and vestibular rehabilitation improved balance in patients with vestibulopathy, but through different mechanisms. Gaze stability is most improved in those who receive vestibular rehabilitation, but Tai Chi training improves whole-body stability and footfall stability without improving gaze stability. In a subsequent study [40], 36 older adults with vestibulopathy were assigned to a 10-week program of vestibular rehabilitation or Tai Chi exercise. The improvements of the Tai Chi group were associated with reorganized neuromuscular pattern in lower extremities, while the vestibular rehabilitation group only had better control of upper body motion to minimize loss of balance. In a recent study, MacIaszek and Osinski [41] assigned 42 older people with dizziness to either a Tai Chi group or a control group. The Tai Chi group practiced a 45-minute exercise twice weekly for 18 weeks and showed significant improvement in up to and go test, forward deflection, backward deflection, and the maximum sway area. 

#### 3.3.4. Prevention of Falls

Balance function begins to decline from middle age, deteriorates in older age, and increases the risk of fall and injury. Suitable exercise training may improve balance function and prevent accidental falls. Recent studies found that Tai Chi has favorable effects on balance function and falls prevention in the elderly. In the Atlanta subgroup of the clinical trial of FICSIT [42], a total of 200 participants were divided into three groups: Tai Chi, balance training, and education. After 15 weeks of training, the fear of falling responses were reduced in the Tai Chi group compared with the education group, and the Tai Chi group reduced the risk of multiple falls by 47.5%. 

Li and colleagues [43] randomly assigned 256 sedentary community-dwelling elderly people to a Tai Chi group or a stretching control group. After 6 months of training, the Tai Chi group showed significantly fewer falls, lower proportions of fallers, and fewer injurious falls than the control group. The risk for multiple falls in the Tai Chi group was 55% lower than that in the control group. In another study, Voukelatos and colleagues [44] reported that 702 community-dwelling older people participated in a Tai Chi class for 16 weeks. The Tai Chi group showed less falls than the control group, and the hazard ratios of falls for the Tai Chi group were 0.72 and 0.67 at 16 weeks and 24 weeks, respectively.

Tai Chi and conventional balance training appear to have similar effects in falls prevention. Huang and colleagues [45] assigned 163 older adults to three interventions groups (education, Tai Chi, and education plus Tai Chi) and one control group. Over a five-month intervention, the education plus Tai Chi group showed a significant reduction in falls and the risk factors of falls. After a one-year follow up, participants who were receiving any one of the interventions showed a reduction in falls compared with the control group. In a recent study, Tousignant and colleagues [46] randomly assigned 152 elderly subjects to a 15-week Tai Chi exercise or conventional physical therapy, and the results showed that both interventions were effective in falls prevention, but Tai Chi showed a better protective effect compared with physical therapy. In a recent randomized trial [47], 684 community-dwelling older adults were assigned to 3 groups: Tai Chi once a week, Tai Chi twice a week, or a low-level exercise program control group for 20 weeks. Over the 17-month period, the rate of falls reduced similarly among the 3 groups (mean reduction of 58%). The results implied that multiple interventions could be used to prevent falls among older adults.

Although many studies have reported favorable effects of Tai Chi on balance and falls prevention, some studies did not find positive evidence. Woo and colleagues [48] randomized 90 men and 90 women into 3 groups (Tai Chi, resistance training, and control), and found no significant changes in balance, muscle strength, and flexibility for either exercise group compared with controls. Logghe and colleagues [49] applied Tai Chi to 269 community-dwelling elderly people with a high risk of falling. The intervention group received Tai Chi training one hour twice weekly for 13 weeks; the control group received usual care. After 12 months, the Tai Chi group did not display lower risk of falls than the control group. 

A meta-analysis including 9 trials (2203 participants) reported that Tai Chi participants had significant improvements in fall rates (2 trials included) and static balance (2 trials included) compared with exercise controls [50]. Compared with nonexercise controls, however, no improvement was found for Tai Chi participants in fall rates (5 trials) or static balance (2 trials), but a significant improvement was found for fear of falling. In a recent meta-analysis, Leung and colleagues [51] reported that Tai Chi was effective in improving balance of older adults, but it may not be superior to other interventions. Although many Tai Chi studies reported positive effects on balance function, the training protocols varied among these studies. In future studies, large randomized trials using a standardized Tai Chi program are required to prove the effect of falls prevention.

### 3.4. Self-Report Physical Function and Quality of Life

Older Tai Chi participants report higher physical function than their sedentary counterparts. Li and colleagues [11] randomly assigned 94 elderly subjects to either a 6-month Tai Chi group (60 min exercise twice weekly) or a wait-list control group. After training, the Tai Chi group experienced significant improvements in all aspects of physical functioning. The Tai Chi group showed improvement in all 6 functional status measures ranging from daily activities such as walking and lifting to moderate-vigorous activities such as running. The results showed that Tai Chi might improve self-reported physical functioning limitations among physically inactive older individuals. In the Atlanta subgroup of the clinical trial of FICSIT [52], elderly subjects were randomly assigned to three groups (Tai Chi, balance training, or exercise education). After 4 months of training, only Tai Chi participants reported improvement in daily activities and overall life.

Tai Chi exercise programs can slow down the decline in health-related quality of life (ADL) among elderly persons. Dechamps and colleagues [53] randomly assigned 160 institutionalized elderly persons to a Tai Chi program (30 min, 4 times/wk), a cognition-action program (30–45 min, 2 times/wk), or a usual-care control group. After 12 months, the Tai Chi and cognition-action groups showed a lesser decline in ADL than the control group. Walking ability and continence were maintained better in the intervention groups than in the control group. The total Neuropsychiatric Inventory score worsened significantly in the control group, while it was unchanged or improved in the intervention groups. 

### 3.5. Psychological Well-Being

Jin [54] reported that Tai Chi practitioners had increased noradrenaline excretion in urine and decreased salivary cortisol concentration. The increase in urine noradrenaline indicated that the sympathetic nervous system is moderately activated during the Tai Chi practice. The decrease in salivary cortisol concentration denoted that Tai Chi is a low-intensity exercise and has similar effects of meditation. The results implied that Tai Chi could reduce tension, depression, and anxiety, and the stress-reduction effect of Tai Chi was similar to walking at speed of 6 km/hr [55]. It is also reported that a 16-week Tai Chi program could reduce mood disturbance and improve general mood in women [56]. For subjects with cardiovascular risk factors, Taylor-Piliae and colleagues [57] have reported that a 60-minute Tai Chi class 3 times weekly for 12 weeks might improve mood state, reduction in anxiety, anger-tension, and perceived stress.

Wang and colleagues [58] reviewed the effect of Tai Chi on psychological profile in 40 studies including 3817 subjects. Twenty-one of 33 randomized and nonrandomized trials reported that regular practice of Tai Chi improved psychological well-being including reduction of stress, anxiety, and depression and enhanced mood. Seven observational studies also demonstrated beneficial effects on psychological health. Jimenez and colleagues [59] reviewed 35 Tai Chi intervention articles in various populations and reported that Tai Chi might provide health benefits to psychological function. In those studies, 9 out of 11 studies confirmed significant improvements in mood and depressive symptoms, 7 out of 8 studies showed reduction in anger and tension, and 6 out of 10 studies displayed improvements in anxiety reduction.

Tai Chi can be applied in patients with depression. In a recent study, Yeung and colleagues [60] randomly assigned 39 patients with major depressive disorders to a 12-week Tai Chi intervention or a wait-list control group. Compared with the control group, the results showed trends toward improvement in positive treatment-response rate and remission rate in the Tai Chi group. 

## 4. Application of Tai Chi in Medicine

An optimal exercise program for adults should address the health-related physical fitness components of cardiorespiratory (aerobic) fitness, muscular strength and endurance, flexibility, body composition, and neuromotor fitness [61]. Previous research suggests that Tai Chi may improve health-related fitness and psychosocial function. Additionally, Tai Chi includes the warm-up and cool-down, stretching exercises, and gradual progression of volume and intensity, and it seems to be helpful to reduce muscular injury and complications. The discussion below will focus on the clinical application in patients with neurological diseases, rheumatological diseases, orthopedic diseases, cardiopumonary diseases and cancers. 

## 5. Tai Chi for Neurological Disease

### 5.1. Stroke

It is estimated that 15 million people experience a stroke worldwide each year. In the United States, about 795,000 people experience a new or a recurrent stroke (ischemic or hemorrhagic) each year [62]. Stroke results in a significant decrease in quality of life, which is determined not only by the neurological deficits but also by impairment of cognitive function. In a recent meta-analysis, Stoller and colleagues [63] reported that stroke patients benefited from exercise by improving peak oxygen uptake and walking distance. Stroke patients usually have impaired balance and motor function; thus, Tai Chi exercise may have potential benefits in stroke rehabilitation. 

Hart and colleagues [64] assigned 18 community-dwelling stroke patients to a Tai Chi group or a control group. The study group practiced Tai Chi one hour twice weekly for 12 weeks, while the control group received conventional physical therapy. After training, the Tai Chi group showed improvement in social and general functioning, whereas the control group showed improvement in balance and speed of walking. The results implied that physical therapy should be served as a main treatment program for stroke patients, but Tai Chi can be used as an alternative exercise program. 

Balance and motor skills in everyday life may benefit when stroke survivors do Tai Chi exercises. Au-Yeung and colleagues [65] randomly assigned 136 stroke patients to a Tai Chi group or a control group practicing general exercises. The Tai Chi group practiced 12 short forms of Tai Chi for 12 weeks. After training, the Tai Chi group showed greater excursion in the center of gravity (COG) amplitude in leaning forward, backward, and toward the affected and nonaffected sides, as well as faster reaction time in moving the COG toward the nonaffected side. The result indicated that Tai Chi training improved standing balance in patients with stroke. 

Tai Chi also shows benefits to the psychological function. Wang and colleagues [66] randomly assigned 34 patients with stroke to Tai Chi exercise or conventional rehabilitation in group sessions once a week for 12 weeks. After training, the Tai Chi group had improvement for sleep quality, general health score, anxiety/insomnia score, and depression score. In a recent study, Taylor-Piliae and Coull [67] recruited 28 stroke patients to participate in a community-based Yang Tai Chi training program. Patients practiced Tai Chi ≥150 minutes/week for 12 weeks. The results showed good satisfaction, and the adherence rates were high (≥92%). There were no falls or other adverse events in the training period. Tai Chi appears to be safe and can be considered as a community-based exercise program for stroke patients.

### 5.2. Parkinson's Disease

Impaired mobility is common among patients with Parkinson's disease (PD). Normal sensorimotor agility and dynamic control are required to maintain balance during motor and cognitive tasks. Gait changes include difficulty in initiating steps, shuffling, and freezing of gait and they are common in patients with PD. Balance difficulties are also prominent during turning and backward walking, and thus patients with PD have high risk of falls [68]. Tai Chi can improve balance, kinesthetic sense, and strength, and hence it may be prescribed as a sensorimotor agility program for patients with PD. 

Li and colleagues [69] designed a Tai Chi program for 17 community-dwelling patients with mild-to-moderate idiopathic PD. Patients participated in a 5-day, 90 min/day training program. At the end of this intervention, the program was well received by all participants with respect to participant satisfaction, enjoyment, and intentions to continue. Furthermore, a significant improvement was observed in 50 ft speed walk, timed up-and-go, and functional reach. The results of this pilot study suggested that even a 5-day Tai Chi program was effective for improving physical function in patients with PD.

In another study [70], 33 patients with PD were randomly assigned to a Tai Chi group or a control group. The Tai Chi group participated in 20 training sessions within 10–13 weeks. After training, the Tai Chi group improved more than the control group on the Berg Balance Scale, the Unified Parkinson's Disease Rating Scale, the timed up-and-go, the tandem stance test, the 6-minute walk, and the backward walking. In a recent study, Li and colleagues [71] randomly assigned 195 patients with PD to one of three groups: Tai Chi, resistance training, or stretching. All patients participated in 60-minute exercise sessions twice weekly for 24 weeks. After training, the Tai Chi group performed better than the other two groups in maximum excursion and in directional control. The Tai Chi group also performed better in strength, functional reach, timed up-and-go, motor scores, and number of falls than the stretching group. Additionally, the Tai Chi group outperformed the resistance-training group in stride length and functional reach. This study revealed that Tai Chi could reduce balance impairments in patients with PD, with improved functional capacity and reduced falls. Tai Chi appears to be a safe and effective exercise for patients with mild-to-moderate PD.

### 5.3. Traumatic Brain Injury

Traumatic brain injury (TBI) is a common disease in the young male population. However, the outcome is disappointing in severely injured patients. Exercise therapy for patients with TBI may improve the motor function and independence. 

Shapira and colleagues [72] reported the application of long-term Tai Chi training in 3 patients with severe TBI. After 2 to 4 years of training, all patients can walk without assistance, rarely fall, and feel more secure while walking. One patient can lead independent daily activities and even return to car driving.

To explore the effects of short-term Tai Chi training in patients with TBI, Gemmell and Leathem [73] assigned 18 patients with TBI to a Tai Chi group (a 6-week course) or a control group. The results showed that Tai Chi was associated with significant improvement on all Visual Analogue Mood Scales scores with decreases in sadness, confusion, anger, tension, and fear and with increases in energy and happiness. However, there were no significant between-group differences in the Medical Outcome Study 36-Item Short-Form Health Survey (SF-36) and Rosenberg Self-Esteem Scale. Recently, Blake and Batson [74] examined the effects of a short-term (eight weeks) Tai Chi Qigong program on 20 patients with TBI. Intervention participants attended a Tai Chi Qigong program for one hour per week, while control participants engaged in nonexercise-based social and leisure activities. After the intervention, mood and self-esteem were improved in the Tai Chi group when compared with controls. There were no significant differences in physical functioning between groups. 

### 5.4. Multiple Sclerosis

Husted and colleagues [75] reported that 19 patients with multiple sclerosis participated in an 8-week Tai Chi program. After training, walking speed increased in 21%, and hamstring flexibility increased in 28%. The results may be attributed to the effect of neuromuscular facilitation during Tai Chi practice. 

## 6. Tai Chi for Rheumatological Disease

There are more than 21% of adults in the United States living with rheumatological diseases, conditions that affect the joints and bones and cause chronic joint pain, swelling, and stiffness [76]. Studies have shown that patients with rheumatological diseases can benefit from Tai Chi exercise. Although Tai Chi is performed in a semisquat posture, joint pain can be prevented because most motions of Tai Chi are performed in a closed kinematic chain and in very slow speed [20]. However, patients with arthropathy should perform Tai Chi in high-squat posture to prevent excessive stress on lower extremities. In a recent review, Tai Chi may modulate complex factors and improve health outcomes in patients with rheumatologic conditions. Tai Chi can be recommended to patients with rheumatoid arthritis, osteoarthritis, and fibromyalgia, as an alternative approach to improve patient's well-being [77]. 

### 6.1. Rheumatoid Arthritis

Rheumatoid arthritis (RA) is a chronic, inflammatory, and systemic disease which affects the musculoskeletal system. In a Cochrane database systemic review including 4 trials and 206 patients with RA [78], Tai Chi does not exacerbate symptoms of RA. In addition, Tai Chi has significant benefits to lower extremity range of motion for patients with RA.

Recently, two studies reported the benefits of Tai Chi for patients with RA. Wang [79] randomly assigned 20 patients with functional class I or II RA to Tai Chi or attention control group. After 12 weeks of training, half of patients in the Tai Chi group achieved a 20% response of the American College of Rheumatology, but no patient in the control group showed improvement. The Tai Chi group had greater improvement in the disability index, the vitality subscale of the SF-36, and the depression index. Similar trends to improvement for disease activity, functional capacity and health-related quality of life were also observed. In another study [80], 15 patients with RA were instructed on Tai Chi exercise twice weekly for 12 weeks. The result showed that the Tai Chi group improved lower-limb muscle function at the end of the training and at 12 weeks of follow up. Patients also experienced improved physical condition, confidence in moving, balance, and less pain during exercise and in daily life. Others experienced stress reduction, increased body awareness, and confidence in moving. These studies indicated that Tai Chi was a feasible exercise modality for patients with RA. 

### 6.2. Ankylosing Spondylitis

 Ankylosing spondylitis (AS) is a chronic inflammatory disease of the axial skeleton with variable involvement of peripheral joints and nonarticular structures. In a recent study [81], Lee and colleagues assigned 40 patients with AS to Tai Chi or control group. The Tai Chi group performed 60 min of Tai Chi twice weekly for eight weeks followed by 8 weeks of home-based Tai Chi. After training, the Tai Chi group showed significant improvement in disease activity and flexibility compared with the control group, and no adverse effects associated with the practice of Tai Chi were reported by the participants. 

### 6.3. Fibromyalgia

Fibromyalgia syndrome is a chronic condition characterized by widespread pain, multiple tender points, nonrestorative sleep, fatigue, cognitive dysfunction, complex somatic symptoms, and poor quality of life [82]. Exercise showed some benefits in the treatment of patients with fibromyalgia. An important study of Tai Chi on fibromyalgia was reported by Wang and colleagues [83]. In this trial, 66 patients with fibromyalgia were randomly assigned to a Tai Chi group or a group that attended wellness education and stretching program. Each session lasted for 60 minutes twice weekly for 12 weeks. After training, the Tai Chi group displayed improvements in the Fibromyalgia Impact Questionnaire (FIQ) total score and SF-36. The SF-36 physical component scores and mental component scores were significantly improved compared with the control group. This study proved that patients with fibromyalgia benefited from Tai Chi training, with no adverse effects. 

Jones and colleagues [84] conducted a randomized controlled trial and assigned 101 patients with fibromyalgia to Tai Chi or education group. The Tai Chi participants practiced modified 8-form Yang-style Tai Chi 90 minutes twice weekly for over 12 weeks. After training, the Tai Chi group demonstrated significant improvements in FIQ scores, pain severity, pain interference, sleep, and self-efficacy for pain control compared with the education group. Functional mobility variables including timed up-and-go, static balance, and dynamic balance were also improved in the Tai Chi group. Tai Chi appears to be a safe and acceptable exercise modality for patients with fibromyalgia.

In a recent study, Romero-Zurita and colleagues [85] reported the effects of Tai Chi training in women with fibromyalgia. Thirty-two women with fibromyalgia attended Tai Chi intervention 3 sessions weekly for 28 weeks. After training, patients improved in pain threshold, total number of tender points, and algometer score. Patients also showed improvement in the 6 min walk, back scratching, handgrip strength, chair stand, chair sit & reach, 8-feet up-and-go, and blind flamingo tests. Additionally, the Tai Chi group improved in the total score and six subscales of FIQ: stiffness, pain, fatigue, morning tiredness, anxiety, and depression. Finally, patients also showed improvement in six subscales in SF-36: bodily pain, vitality, physical functioning, physical role, general health, and mental health. 

## 7. Tai Chi for Orthopedic Disease

### 7.1. Osteoarthritis

Patients with osteoarthritis (OA) show benefits from 6–20 weeks of Tai Chi training. The first randomized trial of Tai Chi and osteoarthritis was conducted by Hartman and colleagues [86]. In this study, 33 older patients with lower extremity OA were assigned to Tai Chi or control group. Tai Chi training included two 1-hour Tai Chi classes per week for 12 weeks. After training, Tai Chi participants experienced significant improvements in self-efficacy for arthritis symptoms, total arthritis self-efficacy, level of tension, and satisfaction with general health status. 

Song and colleagues [87] randomly assigned 72 patients with OA to a Tai Chi group or a control group. The Tai Chi group practiced Sun-style Tai Chi for 12 weeks. After training, the Tai Chi group perceived significantly less joint pain and stiffness and reported fewer perceived difficulties in physical functioning, while the control group showed no change or even deterioration in physical functioning. The Tai Chi group also displayed significant improvement in balance and abdominal muscle strength. In a subsequent study, Song and colleagues [88] reported that Tai Chi could improve knee extensor endurance, bone mineral density in the neck of the proximal femur, Ward's triangle, and trochanter and reduce fear of falling in women with OA. 

Brismée and colleagues [89] reported a randomized controlled trial including 41 elderly patients with OA. Patients were assigned to a Tai Chi or an attention control group. The Tai Chi group participated in six-week Tai Chi sessions, 40 min/session, three times a week, followed by another six weeks of home-based Tai Chi training, and then a six-week follow up detraining period. Subjects in the attention control group attended six weeks of health lectures, followed by 12 weeks of no activity. After six weeks of training, the Tai Chi group showed significant improvements in overall knee pain, maximum knee pain, and the Western Ontario and McMaster Universities Osteoarthritis Index (WOMAC) subscales of physical function and stiffness compared with the baseline. The Tai Chi group reported lower overall pain and better WOMAC physical function than the attention control group, but all improvements disappeared after detraining. The result implies that a short-term Tai Chi program is beneficial for patients with OA, but long-term practice is needed to maintain the therapeutic effect. 

Fransen and colleagues [90] randomly assigned 152 older persons with chronic hip or knee OA to hydrotherapy classes, Tai Chi classes, or a wait-list control group. After 12 weeks of training, both the hydrotherapy group and the Tai Chi group demonstrated improvements for pain, and physical function scores and achieved improvements in the 12-Item Short From Health Survey (SF-12) physical component summary score. This study revealed that Tai Chi and hydrotherapy can provide similar benefits to patients with chronic hip or knee OA. 

In a randomized controlled trial conducted by Wang and colleagues [91], 40 patients with OA were assigned to Tai Chi group or attention control group. The Tai Chi group practiced 10 modified Yang Tai Chi postures twice weekly for 12 weeks. After training, the Tai Chi group significantly improved in WOMAC pain, WOMAC physical function, patient and physician global visual analog scale, chair stand time, Center for Epidemiologic Studies Depression Scale, self-efficacy score, and SF-36 physical component summary. The result showed that Tai Chi reduces pain and improves physical function, self-efficacy, depression and health-related quality of life for patients with knee OA. 

In a recent randomized controlled study [92], 58 community-dwelling elderly patients with knee OA and cognitive impairment were assigned to a Tai Chi (20-week program) or a control group. After training, the Tai Chi group showed significant improvement in WOMAC pain, physical function, and stiffness score than the control group. The result showed that practicing Tai Chi was effective in reducing pain and stiffness in patients with knee OA and cognitive impairment. 

Tai Chi is also beneficial to gait kinematics for the elderly with knee OA. Shen and colleagues [93] applied Tai Chi on 40 patients with knee OA. Patients participated in 6-week Tai Chi training (1 hour/session, 2 sessions/week). After 6 weeks of Tai Chi exercise, patient's stride length, stride frequency, and gait speed were significantly increased, and knee pain was decreased. 

### 7.2. Osteoporosis

Osteoporosis is the most common metabolic bone disorder, and it is estimated that 44 million individuals in the United States over the age of 50 years have osteoporosis or low bone mass [94]. Exercise is an effective therapy to prevent or delay the development of osteoporosis. Qin and colleagues [95] reported that Tai Chi participants had significantly higher bone mineral density (BMD) than the controls in the lumbar spine, the proximal femur, and the ultradistal tibia. The follow up measurements showed generalized bone loss in both groups, but the quantitative computed tomography revealed significantly reduced rate of bone loss in trabecular BMD of the ultradistal tibia and of the cortical BMD of the distal tibial diaphysis. In a subsequent study, Chan and colleagues [96] randomly assigned 132 healthy postmenopausal women to Tai Chi or sedentary control group. The Tai Chi group practiced Tai Chi 45 minutes a day, 5 days a week for 12 months. At 12 months of training, BMD measurements revealed a general bone loss in both Tai Chi and control subjects at lumbar spine, proximal femur, and distal tibia, but with a slower rate in the Tai Chi group. A significant 2.6- to 3.6-fold retardation of bone loss was found in both trabecular and cortical compartments of the distal tibia in the Tai Chi group as compared with the controls. 

In a recent trial, Wayne and colleagues [97] reported the application of Tai Chi in 86 postmenopausal osteopenic women aging 45–70 years. Women were assigned to either 9 months of Tai Chi training plus usual care or usual care alone. Protocol analyses of femoral neck BMD changes were significantly different between Tai Chi and usual care-group. Changes in bone formation markers and physical domains of quality of life were more favorable in the Tai Chi group. 

### 7.3. Low-Back Pain

Chronic low-back pain (LBP) is prevalent in the general population, and exercise therapy is among the effective interventions showing small-to-moderate effects for patients with LBP. In a recent randomized trial [98], 160 volunteers with chronic LBP were assigned either to a Tai Chi group or to a wait-list control group. The Tai Chi group participated in 18 training sessions (40 minutes per session over a 10-week period), and the wait-list control group continued with usual healthcare. After training, the Tai Chi group reduced bothersomeness of back symptoms by 1.7 points on a 0–10 scale, reduced pain intensity by 1.3 points on a 0–10 scale, and improved self-report disability by 2.6 points on the 0–24 Roland-Morris Disability Questionnaire scale. Though the improvements were modest and most of the patients were not “completely recovered”, the results showed that a 10-week Tai Chi program provides benefits for pain reduction considered clinically worthwhile for those experiencing chronic LBP.

### 7.4. Musculoskeletal Disorder

Musculoskeletal disorder is a leading cause of work disability and productivity losses in industrialized nations. Tai Chi can be used as a simple, convenient workplace intervention that may promote musculoskeletal health without special equipment. A recent study applied Tai Chi to female computer users [99], and 52 subjects participated in a 50-minute Tai Chi class per week for 12 weeks. The results showed significant improvement in heart rate, waist circumference, and hand-grip strength. It implied that Tai Chi was effective in improving musculoskeletal fitness. 

In chronic muscular pain, such as tension headache, Tai Chi also shows some benefits. Abbott and colleagues [100] randomly assigned 47 patients with tension headache to either a 15-week Tai Chi program or a wait-list control group. The SF-36 and headache status were obtained at baseline and at 5, 10, and 15 weeks during the intervention period. After training, the results revealed significant improvements in favor of Tai Chi intervention for the headache status score and the subsets of health-related quality of life, including pain, energy/fatigue, social functioning, emotional well-being, and mental health summary scores. 

## 8. Tai Chi for Cardiovascular Disease

In the United States, the relative rate of death attributable to cardiovascular disease (CVD) declined by 32.7% from 1999 to 2009; however, CVD still accounted for 32.3% of all deaths in 2009 [62]. Exercise training is the core component of cardiac rehabilitation (CR) for patients with coronary heart disease (CHD). Tai Chi may be used in CR programs because its exercise intensity is low to moderate, and it can be easily implemented in communities. In a recent study, Taylor-Piliae and colleagues [101] reported a study that included 51 cardiac patients who participated in an outpatient CR program. Patients were assigned to attend a group practicing Tai Chi plus CR or a group to attend CR only. After rehabilitation, subjects attending Tai Chi plus CR had better balance, perceived physical health, and Tai Chi self-efficacy compared with those attending CR only. 

### 8.1. Cardiovascular Risk Factors

#### 8.1.1. Hypertension

Hypertension is the most prevalent form of CVD affecting approximately 1 billion patients worldwide. In the United States, about one in three adults has hypertension [62]. Hypertension is a major risk factor for coronary artery disease, heart failure, stroke, and peripheral vascular disease. Regular exercise and lifestyle change are the core of current recommendations for prevention and treatment of hypertension. Systemic review of randomized clinical trials indicated that aerobic exercise significantly reduced BP, and the reduction appears to be more pronounced in hypertensive subjects [102, 103].

Previous studies have shown that 6- to 12-week Tai Chi training programs might decrease systolic and diastolic BP at rest or after exercise, and hypertensive patients exhibit the most favorable improvement [104–108]. In a recent systemic review, Yeh and colleagues [109] analyzed 26 studies and found positive effect of Tai Chi on blood pressure. In patients with hypertension, studies showed that Tai Chi training might decrease systolic BP (range: −7 to −32 mm Hg) and diastolic BP (−2.4 to −18 mm Hg). In studies for noncardiovascular populations or healthy patients, the decreases ranged from −4 to −18 mm Hg in systolic BP and from −2.3 to −7.5 mm Hg in diastolic BP. For patients with acute myocardial infarction (AMI), both Tai Chi and aerobic exercise were associated with significant reductions in systolic BP, but diastolic BP was decreased in the Tai Chi group only. 

#### 8.1.2. Diabetes Mellitus

Diabetes mellitus is a fast growing risk factor for cardiovascular disease. Estimated 19.7 million American adults have diabetes, and the prevalence of prediabetes in the US adult population is 38% [62]. Previous studies have shown that exercise has benefits for those who have diabetes or impaired glucose tolerance [110–112]. In the Da Qing Diabetes Prevention Study [113] for people with impaired glucose tolerance, lifestyle intervention groups (diet and exercise) displayed a 43% lower incidence of diabetes than the control group over the 20-year follow up period. 

Several studies have shown the benefits of Tai Chi for diabetic patients. In a pilot study for 12 patients with diabetes, Wang [114] reported that an 8-week Tai Chi program could decrease blood glucose. Additionally, high- and low-affinity insulin receptor numbers and low-affinity insulin receptor-binding capacity were increased. For obese diabetic patients, Chen and colleagues reported that 12 weeks of Chen Tai Chi training induced significant improvement in body mass index, triglyceride (TG), and high-density lipoprotein cholesterol (HDL-C) [115]. In addition, serum malondialdehyde (oxidative stress indicator) and C-reactive protein (inflammation indicator) decreased significantly. 

In diabetic patients complicated with peripheral neuropathy, Ahn and Song reported that Tai Chi training one hour twice per week for 12 weeks improved glucose control, balance, neuropathic symptoms, and some dimensions of quality of life [116]. A recent study reported that a 12-week Tai Chi program for diabetic patients obtained significant benefits in quality of life [117]. After training, the Tai Chi group revealed significant improvements in the SF-36 subscales of physical functioning, role physical, bodily pain, and vitality. 

#### 8.1.3. Dyslipidemia

Dyslipidemia, or abnormalities in blood lipid and lipoprotein, is a major risk factor of cardiovascular disease. In the United States, 26.0% of adults had hypercholesterolemia during the period from 1999 to 2006, and approximately 27% of adults had a triglyceride level ≥150 mg/dL during 2007 to 2010 [62]. The prevalence of dyslipidemia increases with age and westernized lifestyle, but regular exercise may ameliorate the trend toward abnormal blood lipid profile. A meta-analysis of 31 randomized controlled trials with exercise training reported a significant decrease in total cholesterol (TC), low-density lipoprotein cholesterol (LDL-C), and triglyceride, and an increase in HDL-C [118]. 

Tsai and colleagues [107] randomly assigned 88 patients to Tai Chi or sedentary control group. After 12 weeks of classical Yang Tai Chi training, TC, TG, and LDL-C decreased by 15.2, 23.8, and 19.7 mg/dL, respectively, and HDL-C increased by 4.7 mg/dL. By contrast, Thomas and colleagues [119] reported no significant change in TC, TG, LDL-C, and HDL-C after 12 months of Tai Chi training. This may be attributed to differences in baseline lipid concentrations, training amount and intensity, changes in body composition, or the adjunctive interventions such as diet or lipid-lowering agents. 

**Figure 4:**
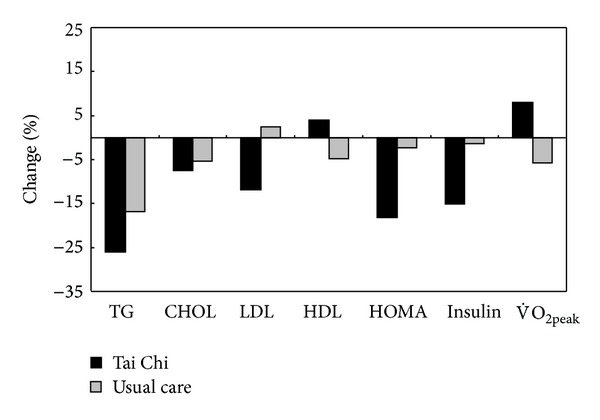
Changes of peak $\dot{\text{V}}{\text{O}}_{2}$ and cardiovascular risk factors after 1 year of training in patients with dyslipidemia (Tai Chi group versus usual-care group).

In a recent study, Lan and colleagues [120] assigned 70 dyslipidemic patients to a 12-month Yang Tai Chi training group or the usual-care group. After training, the Tai Chi group showed a significant decrease of 26.3% in TG (from 224.5 ± 216.5 to 165.9 ± 147.8 mg/dL), 7.3% in TC (from 228.0 ± 41.0 to 211.4 ± 46.5 mg/dL), and 11.9% in LDL-C (from 134.3 ± 40.3 to 118.3 ± 41.3 mg/dL), whereas the HDL-C did not increase significantly. In addition, the Tai Chi group also showed a significant decrease in fasting insulin and a decrease in homeostasis model assessment of insulin resistance (HOMA) index, which is suggestive of improved insulin resistance (Figure 4). 

### 8.2. Acute Myocardial Infarction

Acute myocardial infarction is the most common cause of mortality in patients with cardiovascular disease, but exercise can significantly reduce the mortality rate in patients with AMI. A recent Cochrane review [121] involved in 47 studies randomizing 10,794 patients with AMI to exercise-based cardiac rehabilitation or usual care. Patients receiving exercise training reduced a 13% of risk for total mortality, a 26% of risk for cardiovascular mortality, and a 31% of risk for hospital admissions. Channer and colleagues [104] randomized 126 patients with AMI to Tai Chi, aerobic exercise, or nonexercise support group. The Tai Chi and the aerobic exercise group participated in an 8-week training program, attended twice weekly for three weeks, and then once weekly for five weeks. The results displayed that Tai Chi was effective for reducing systolic and diastolic BP and that it was safe for patients after AMI.

### 8.3. Coronary Artery Bypass Grafting

Lan and colleagues [122] assigned 20 patients after coronary artery bypass grafting surgery (CABG) to classical Yang Tai Chi program or maintenance home exercise. After 12 months of training, the Tai Chi group showed significant improvements of oxygen uptake at the peak exercise and the ventilatory threshold. At the peak exercise, the Tai Chi group showed 10.3% increase in $\dot{\text{V}}{\text{O}}_{2}$, while the control group did not show any improvement. Furthermore, the Tai Chi group increased 17.6% in $\dot{\text{V}}{\text{O}}_{2}$ at the ventilatory threshold, while the control group did not display significant change. The result showed that Tai Chi was safe and had benefits in improving functional capacity for patients after CABG.

### 8.4. Congestive Heart Failure

Congestive heart failure (CHF) is characterized by the inability of the heart to deliver sufficient oxygenated blood to tissue. CHF results in abnormalities in skeletal muscle metabolism, neurohormonal responses, vascular and pulmonary functions. In 2009, heart failure was the underlying cause in 56,410 of those deaths in the United States [62]. Exercise training improves functional capacity and symptoms in patients with CHF, and the increase in exercise tolerance may be attributed to increased skeletal muscle oxidative enzymes and mitochondrial density. Previous studies have shown that low-intensity Tai Chi training benefited patients with CHF [123–128]. In a study by Barrow and colleagues [123], 52 patients with CHF were randomized to Tai Chi or standard medical care group. The Tai Chi group practiced Tai Chi twice a week for 16 weeks. After training, the Tai Chi group did not show significant increase in exercise tolerance, but they had improvement in symptom scores of heart failure and depression scores compared with the control group. Yeh and colleagues [124, 125] also reported that a 12-week Tai Chi training in patients with CHF improved quality of life, sleep quality, and 6-minute walking distance and decreased serum B-type natriuretic peptide (BNP). BNP is produced by ventricular cardiomyocytes and is correlated with left ventricular dysfunction. In a recent study, Yeh and colleagues [126] randomized 100 patients with systolic heart failure into a Tai Chi group or a control group. Tai Chi participants practiced 5 basic simplified Yang Tai Chi movements twice weekly, while the control group participated in an education program. After 12 weeks of training, the Tai Chi group displayed greater improvements in quality of life, exercise self-efficacy, and mood. For patients with CHF, low-intensity exercise such as simplified Tai Chi may increase the acceptance. Interval training protocol by using selected Tai Chi movements is suitable for patients with very low endurance. 

Tai Chi can combine endurance exercise to improve functional capacity. Caminiti and colleagues [127] enrolled 60 patients with CHF and randomized them into a combined training group performing Tai Chi plus endurance training, and an endurance training group. After 12 weeks of training, 6-minute walking distance increased in both groups, but the combined training group showed more improvement than the endurance training group. Systolic BP and BNP decreased in the combined training group compared with the endurance training group. Additionally, the combined training group had a greater improvement in physical perception and peak torque of knee extensor compared with the endurance training group. 

The left ventricle ejection fraction is found to be preserved in about half of all cases of heart failure. Patients with heart failure with preserved ejection fraction (HFPEF) appear to be older and are more likely to be females, have a history of hypertension, and have less coronary artery diseases [128]. Yeh and colleagues [129] recently used Tai Chi in the treatment of patients with HFPEF, and 16 patients were randomized into 12-week Tai Chi or aerobic exercise. Change in $\dot{\text{V}}{\text{O}}_{2\text{peak}}$ was similar between groups, but 6-minute walking distance increased more in the Tai Chi group. Both groups had improved Minnesota Living With Heart Failure scores and self-efficacy, but the Tai Chi group showed a decrease in depression scores in contrast to an increase in the aerobic exercise group. In patients with HFPEF, the Tai Chi group displayed similar improvement as the aerobic exercise group despite a lower aerobic training workload. 

## 9. Tai Chi for Pulmonary Disease

Chronic obstructive pulmonary disease (COPD) is the fourth leading cause of mortality in the United States. Patients with COPD are at risk for low levels of physical activity, leading to increased morbidity and mortality [130]. The effectiveness of exercise training in people with COPD is well established. However, alternative methods of training such as Tai Chi have not been widely evaluated. 

Chan and colleagues [131] have evaluated the effectiveness of a 3-month Tai Chi Qigong (TCQ) program in patients with COPD. 206 patients with COPD were randomly assigned to three groups (TCQ, exercise, and control). Patients in the TCQ group participated in a TCQ program, including two 60-minute sessions each week for 3 months; patients in the exercise group practiced breathing exercise combined with walking. After training, the TCQ group showed greater improvements in the symptom and activity domains. In addition, the forced vital capacity, forced expiratory volume in the first second, walking distance, and exacerbation rate were improved in the TCQ group [132]. 

In a pilot study conducted by Yeh and colleagues [133], 10 patients with moderate-to-severe COPD were randomized to 12 weeks of Tai Chi plus usual care or usual care alone. After training, there was significant improvement in Chronic Respiratory Questionnaire score in the Tai Chi group compared with the usual-care group. There were nonsignificant trends toward improvement in 6-minute walk distance, depression scale, and shortness of breath score. 

In a recent study, Leung and colleagues [134] examined the effect of short-form Sun-style Tai Chi training in people with COPD. Forty-two participants were randomly allocated to Tai Chi or usual-care control group. Participants in the Tai Chi group trained twice weekly for 12 week, and the exercise intensity of Tai Chi was 53% ± 18% of oxygen uptake reserve. Compared with the control, Tai Chi significantly increased endurance shuttle walk time, reduced medial-lateral body sway in semitandem stand, and increased total score on the Chronic Respiratory Disease Questionnaire. 

## 10. Tai Chi for Cancer

Cancer is a leading cause of death worldwide. Exercise therapy is a safe adjunct therapy that can mitigate common treatment-related side effects among cancer patients [135]. Additionally, exercise has beneficial effects on certain domains of health-related quality of life (QOL) including physical functioning, role functioning, social functioning, and fatigue [136]. Tai Chi has been reported to be beneficial for physical, emotional, and neuropsychological functions in patients with breast cancer [137–140], lung cancer [141], and gastric cancer [142]. 

 In a recent randomized trial, 21 breast cancer survivors were assigned to Tai Chi or standard support therapy (controls), and patients in the exercise group practiced Tai Chi three times per week and 60 minutes per session for 12 weeks [140]. After training, the Tai Chi group improved in total QOL, physical functioning, physical role limitations, social functioning, and general mental health. Tai Chi may improve QOL by regulating inflammatory responses and other biomarkers associated with side effects from cancer and its treatments. By contrast, a recent meta-analysis did not show convincing evidence that Tai Chi is effective for supportive breast cancer care [143]. Most Tai Chi studies are focused on QOL of breast cancer survivors; however, the positive results must be interpreted cautiously because most trials suffered from methodological flaws such as a small-sample size and inadequate study design. Further research involving large number of participants is required to determine optimal effects of Tai Chi exercise for cancer patients.

## 11. Future Research of Tai Chi 

The training effect of an exercise program depends on its exercise mode, intensity, frequency, and duration. Although previous studies have shown that Tai Chi has potential benefits, most of the studies have limitations in study design, such as (1) a small-sample size, (2) nonrandomized trials, (3) lack of training intensity measurement, and (4) significant differences in training protocols. In future research, a randomized controlled trial with standardized training protocol should be utilized according to the principles of exercise prescription. Tai Chi participants usually need 12 weeks of training to familiarize the movements. During the familiarization phase, the exercise intensity and amount of training are inconsistent. Therefore, a suitable training program should take at least 6 months of training. Additionally, heart rate monitoring in selected individuals is recommended to determine the exercise intensity of Tai Chi, and the suitable duration of training is 40 to 60 minutes including warm-up and cool-down. 

## 12. Conclusion

Tai Chi is a Chinese traditional conditioning exercise that integrated breathing exercise into body movements. This literature paper reveals that Tai Chi has benefits in health promotion and has potential role as an alternative therapy in neurological, rheumatological, orthopedic, and cardiopulmonary diseases. There are several reasons to recommend Tai Chi as an exercise program for healthy people and patients with chronic diseases. First, Tai Chi does not need special facility or expensive equipment, and it can be practiced anytime and anywhere. Second, Tai Chi is effective in enhancing aerobic capacity, muscular strength, and balance and in improving cardiovascular risk factors. Third, Tai Chi is a low-cost, low- technology exercise, and it can be easily implemented in the community. It is concluded that Tai Chi is effective in promoting health, and it can be prescribed as an alternative exercise program for patients with certain chronic diseases.
